# The impact of Pain-related emotions on migraine

**DOI:** 10.1038/s41598-020-80094-7

**Published:** 2021-01-12

**Authors:** Seonghoon Kim, Dae-Woong Bae, Sang-Gue Park, Jeong-Wook Park

**Affiliations:** 1 Department of Neurology, Uijeongbu St. Mary’s Hospital, College of Medicine, The Catholic University of Korea, Seoul, Republic of Korea; 2 Department of Neurology, St. Vincent’s hospital, College of Medicine, The Catholic University of Korea, Seoul, Republic of Korea; 3 Department of Applied Statistics, Chung-Ang University, Seoul, Republic of Korea

**Keywords:** Migraine, Psychiatric disorders

## Abstract

The response to pain is highly individual and can be influenced by complex emotional perception. This study aims to investigate the status of the pain-related emotional response, and the influence on headache characteristics and disability in migraine. We studied the pain-related emotional response in 145 consecutive migraine patients using the Pain Anxiety Symptoms Scale (PASS), the Pain Catastrophizing Scale (PCS), and the Pain Sensitivity Questionnaire (PSQ) and compared them with 106 healthy controls. We investigated the relationship between emotional factors and migraine characteristics. The effect of pain-related emotion on migraine-related disability assessed with the Headache Impact Test-6 (HIT-6) and the Migraine Disability Assessment (MIDAS). Migraine patients showed significantly higher scores on total PASS (*p* < 0.001), PCS (*p* < 0.001) and PSQ (*p* = 0.002) compared to the healthy controls. The HIT-6 was weakly correlated with PASS (r = 0.390, *p* < 0.001) and PCS (r = 0.354, *p* < 0.001). PASS-Total (*p* = 0.001), headache frequency (*p* = 0.003), and HADS-Anxiety (*p* = 0.028) were independent variables associated with HIT-6. Headache frequency (*p* < 0.001) was an independent variable associated with MIDAS. The structural equation model indicated that headache severity has direct loading on emotion and subsequently influenced migraine-related disability. Disability has a significant effect on the frequency of abortive medication use. Migraine patients have altered emotional responses to pain perception. Pain-related anxiety made an important contribution to headache-related disability. The present results suggest that the management of disability by considering various pain-related emotional factors may be necessary for the therapeutic aspects of migraine.

## Introduction

Migraine is one of the most common headache disorders, with a worldwide prevalence ranging from 5 to 12%. The World Health Organization (WHO) has recognized migraine as an urgent public health priority and listed it as a major leading cause of disability^1^. Despite proper medical treatment, some migraine patients continue to experience disabling headaches, contributing to considerable social and economic loss^2^. The perception of pain and functional impairment related to migraine attacks appears to have an individual diversity, which surpasses the contributory role of objective factors associated with headache characteristics, including the frequency and severity of headache attacks^3^.

The interpretation and response to pain are highly subjective and vary widely depending on the individual perception of pain^4^. As a result of inappropriate cognitive processes, patients could suffer from pain beyond the basis of physical conditions^5,6^. Enhanced pain perception or sensitivity might be a significant risk factor in the chronicity of pain^4^. Fear or anxiety about pain related to the excessive prediction of future pain in some patients contributes significantly to their somatic symptoms and disability^7,8^. The belief that current pain will persist or blind faith appears to have a negative impact on coping and compliance, regarding pain as mysterious and inexplicable are associated with poor outcome, psychological distress, and somatization^9^.

Headache patients with a lack of self-efficacy awareness and inadequate response strategies were more prone to poor outcomes in overall disability^10^. In terms of experiencing recurrent headaches, it is theoretically plausible that the emotional properties of pain might play a role in influencing the functional outcome, including disability in migraine patients.

Several measurement tools have been proposed and widely used to evaluate pain-related emotional aspects. The Pain Anxiety Symptoms Scale (PASS) is a questionnaire to measure pain-related anxiety and physical symptoms^7,11,12^. The Pain Catastrophizing Scale (PCS), which mainly evaluated individuals’ tendencies to focus on the pain, and the Pain Sensitivity Questionnaire (PSQ), which assesses pain sensitivity of various pain stimulation, are also being used as clinical evaluation tools^13,14^. The present study investigates the impact of the pain-related emotional response on pain perception and disability in migraine, by using PASS, PCS, and PSQ.

**Figure 1:**
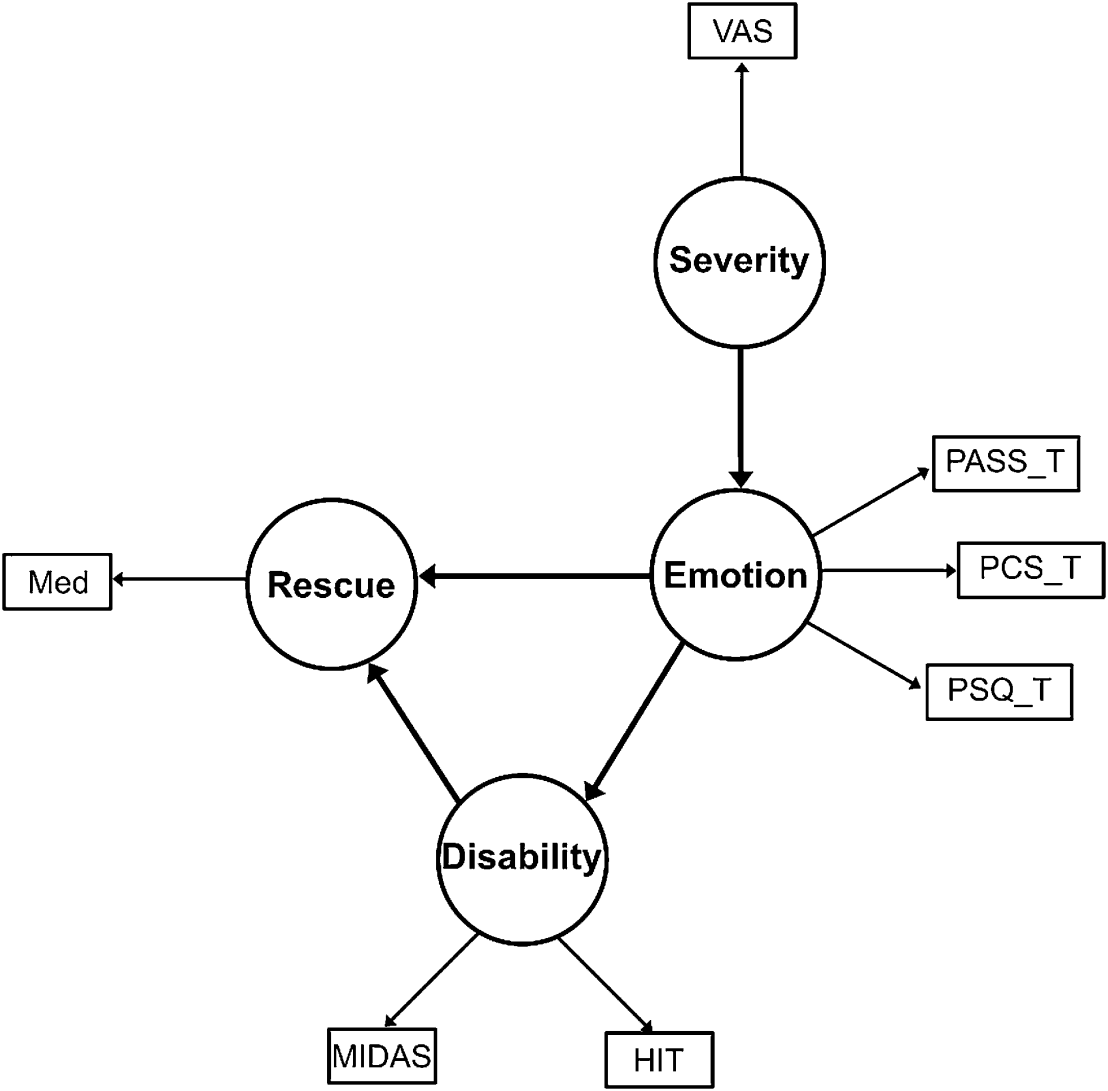
Path illustration of the hypothesized model. *Note* PASS_T, PASS-Total; PCS_T, PCS-Total, PSQ_T, PSQ-Total; Severity, headache severity; VAS, visual analog scale; Rescue, abortive use; Med, frequency of abortive medication use.

The severe headache enhances the fear of pain and avoidance behavior^3^. The pain related-emotional factors aggravate the headache disability^3,15^. Base on these findings, we hypothesized that headache severity might aggravate the pain-related emotional factor, and pain-related emotional factors might influence headache-related disability and behavior. We set the structural equation model (Fig. 1) with the four-latent variables (Emotion, disability, Severity, and Rescue). The total score of PASS, PCS, and PSQ used as measured variables of emotion. The HIT and MIDAS used as measurable variables of disability.

## Materials and methods

### Participants and baseline evaluation

Participants who met the inclusion criteria were enrolled from the neurology outpatient clinics of two hospitals. We use the smartphone headache diary system (SHD) for enrollment of migraine patients. The SHD system collects headache frequency, duration, intensity, disability, and other clinical data from migraine participants^16,17^. All migraine participants use the SHD system for more than three months. The diagnosis of the headache disorder was based on the third edition beta version of the International Classification of Headache Disorder (ICHD-3)^18^. Inclusion criteria of migraine patients were as follows: (1) age between 19 and 75 years and with a diagnosis of migraine, (2) at least two headache-days per month, and (3) stable headache characteristics for at least one year before study entry. We excluded patients if they presented any of the following: (1) secondary headache disorders, (2) any systemic medical disease associated with chronic pain (e.g., rheumatoid arthritis, fibromyalgia, cervical radiculopathy, and cancer-related pain), (3) any recent painful situation except headache (abdominal pain, trauma, back pain, and other joint pain), and (4) any psychological disease-associated anxiety or depression.

We recruit a healthy control group aged between 19 and 75 without a history of migraine and another headache disorder. Exclusion criteria of healthy control follows: (1) any headache or painful situation for the recent three months, (2) history of other systemic medical diseases, chronic pain disorder, and trauma (3) any psychological disease-associated anxiety or depression.

Various factors, such as demographics, headache characteristics (duration of illness, attack frequency, location, quality of pain), and abortive medications were recorded. The psychological status was recorded using the Hospital Anxiety and Depression Scale (HADS). The Korean version of the Migraine Disability Assessment Scale (MIDAS) and the Korean version of Headache Impact Test-6 (HIT-6) were recorded to evaluate the baseline impact or disability related to migraine.

We determined the sample size by using G*power software (version 3.1.9.6)^19,20^. The previous study reported the PASS of the clinical and nonclinical group^21^. Between migraine and healthy control, the calculated sample size (two-tailed, alpha 0.05, power 0.95) for the t-test was 52. For multiple regression analysis, the calculated sample size of migraine group was 142 (alpha 0.05, power 0.95, number of predictor 7) using the previous result^22^.

The protocol of this study was reviewed and approved by the Institutional Review Board/Ethics Committee of the Catholic University of Korea College of Medicine (IRB approval number: 2015-0194-0003). The participants received a detailed explanation of the aim and procedure of the study and provided written informed consent. This study was conducted in ethical principles, as described in the Declaration of Helsinki.

### Measures of PASS, PCS, and PSQ

Included patients and healthy controls were requested to complete the Korean version of the PASS, PCS, and PSQ. The migraine patient group completed the questionnaire on the interictal stage without headache pain. All of the Korean versions have had their validity and reliability levels formerly verified^11,23,24^. The PASS is used to measure pain-related anxiety. It consists of 20 items measured on a six-point scale from 0 (never) to 5 (always). There are four sub-scales with five items. These are fear of pain (FP), cognitive anxiety (CA), somatic anxiety (SA), and escape and avoidance (EA)^7^. We used the total score and the sum of each sub-scale as clinical outcomes. The PCS is a 13 items questionnaire to measure levels of pain catastrophizing. Each item is rated on a five-point scale, from 0 (not at all) to 4 (all the time). There are three sub-scales includes Rumination (RM: 4 items), Magnification (MG: 3 items), and Helplessness (HP: 6 items)^25^. We used the total score of all 13 items and the sum of the scores of each sub-item as clinical outcomes. The PSQ assumes various daily pain situations and is based on a self-rating of pain severity on different body parts. It is composed of 17 items, and each item is rated on an 11-point scale, from 0 (does not seem painful at all) to 10 (seems like the most excruciating pain imaginable). There are two sub-scales with seven items that include Minor Pain (min) and Moderate Pain (mod)^26^. The PSQ-Total score was calculated as the average of the 14 items. PSQ-minor and moderate score were calculated as the average rating of seven items^26^.

### Statistical analysis

The total and sub-domain scores of PASS, PCS, and PSQ were compared between migraine patients and healthy control using an independent-sample t-test. In the migraine group, the Pearson correlation analysis was used to evaluate the association between PASS, PCS, PSQ, and migraine characteristics. The multiple regression analysis was performed in the migraine group to determine independent factors associated with each migraine-related disability (HIT and MIDAS). The migraine characteristics (duration of illness, duration of attack, headache pain intensity, headache frequency) and total score of pain-related emotional factors (PASS, PCS, and PSQ) are used as independent variables and stepwise selection methods used in multiple regression analysis. These analyses were correlated in order to ensure that multicollinearity did not exist. *p* values of less than 0.05 were deemed to be statistically significant. We use a structural equation model to evaluate the relationship between headache disability, severity, emotional factors, and abortive medication use. To evaluate the fit of our model, the absolute fit index (χ^2^), the comparative fit index (CFI), the Tucker–Lewis index (TLI), normed fit index (NFI), and the root mean square error approximation (RMSEA) were used. All of the statistical analyses were performed using R (version 3.6.2).

## Results

### Demographics

**Figure 2:**
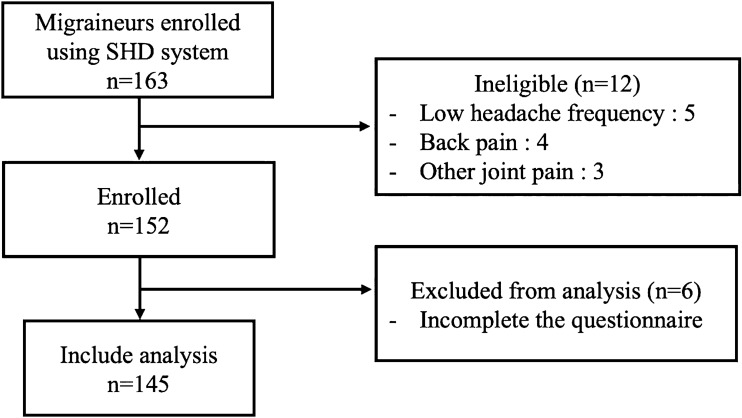
Flow chart depicting the migraine participation by using smartphone headache diary (SHD).

For this study, we enrolled 163 migraine participants. 18 individuals withdrew from the study due to the low frequency of headache attack, another painful situation, and an incomplete questionnaire. Finally, 145 migraine participants and 106 healthy control completed the study (Fig. 2).

The mean age of the migraine group was 38.9 ± 10.1 years, and 125 participants (86.2%) were women. The mean age of healthy control 39.5 ± 12.2 years, and 82 participants (74.5%) were women. There were no statistically significant differences between the groups for all demographic variables (Table 1).

**Table 1:** Demographics of migraine patients and healthy control.

	Migraine patients (N = 145)	Healthy control (N = 106)	*p* value
Age (year)	38.9 ± 10.1	39.2 ± 12.1	0.846
Sex (female)	125 (86.2%)	82 (77.4%)	0.098
Marriage (married)	96 (66.2%)	78 (73.6%)	0.266
Village: city/rural (city)	131 (90.3%)	99 (93.4%)	0.528

*p* values were determined by independent t-tests for numerical variables and chi-square tests for categorical variables.

### Comparison of the pain-related emotional factors between migraine patients and the healthy control

The score of the PASS-Total in migraine patients was significantly higher than the control group (47.4 ± 17.2 vs 19.7 ± 16.3, *p* < 0.001). In sub-domain analysis, PASS-FP (9.4 ± 5.5 vs 4.0 ± 3.9, *p* < 0.001), PASS-CA (15.1 ± 5.8 vs 6.4 ± 5.4, *p* < 0.001), PASS-SA (8.7 ± 5.1 vs 3.0 ± 3.7, *p* < 0.001), and PASS-EA (14.3 ± 5.5 vs 6.3 ± 4.7, *p* < 0.001) were all significantly higher than the control group.

The score of PCS-Total in migraine patients was significantly higher than the control group (24.4 ± 14.1 vs 7.7 ± 8.8, *p* < 0.001). In sub-domain analysis, PCS-RM (10.1 ± 4.5 vs 3.3 ± 3.9, *p* < 0.001), PCS-MG (5.2 ± 2.0 vs 2.0 ± 2.2, *p* < 0.001) and PCS-HP (9.1 ± 7.1 vs 2.4 ± 2.8, *p* < 0.001) were all significantly higher than the control group.

**Figure 3:**
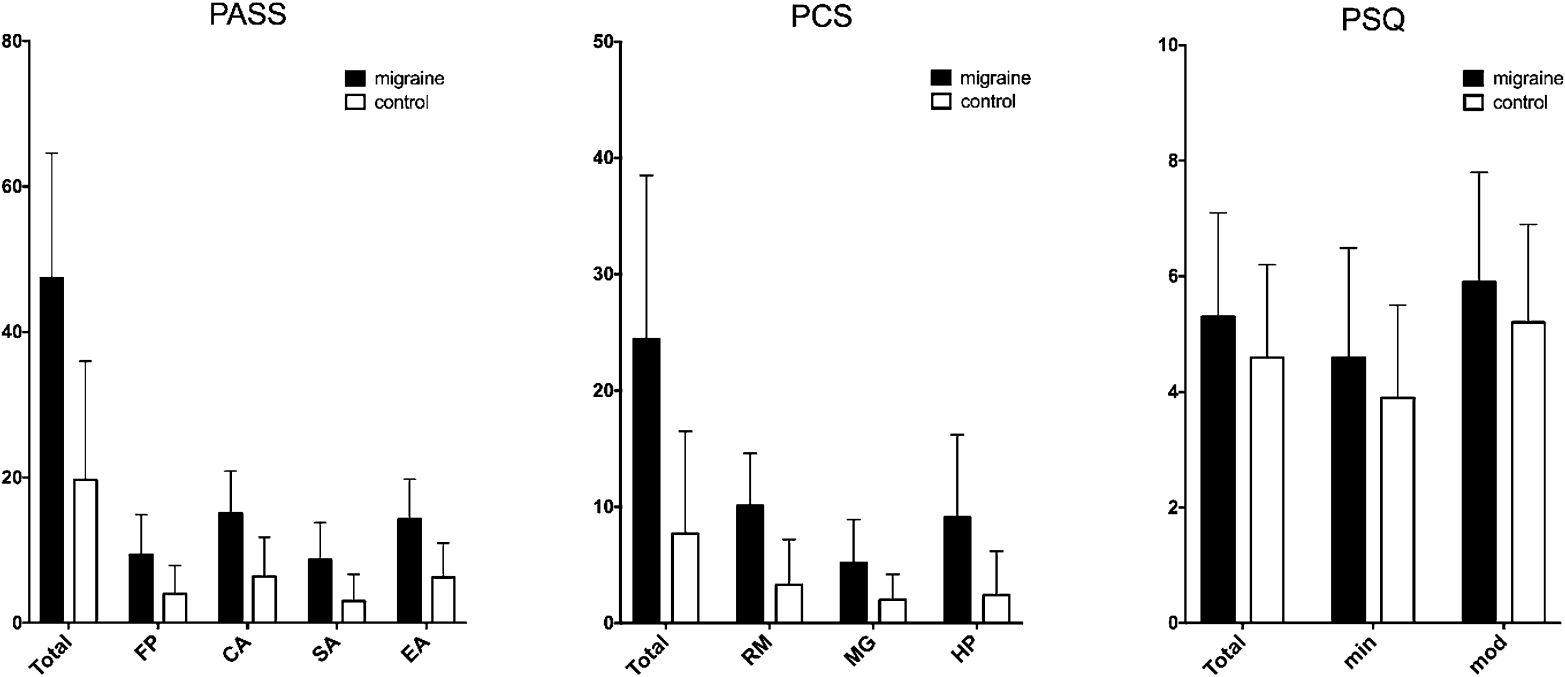
The graphs show a statistically significant difference in emotional factors of pain perception between migraine patients with migraine and healthy controls (*p* < 0.05).

The score of PSQ-Total in migraine patients was significantly higher than in the control group (5.3 ± 1.8 vs 4.6 ± 1.6, *p* = 0.002). In sub-domain analysis, both PSQ-min (4.6 ± 1.9 vs 3.9 ± 1.6, *p* = 0.002) and PSQ-mod (5.9 ± 1.9 vs 5.2 ± 1.7, *p* = 0.002) were significantly higher than the control group (Fig. 3).

### The correlation between pain-related emotion, headache characteristics and disability in migraine patients

Headache pain intensity measured by the visual analogue scale (VAS). Headache pain intensity showed a weak correlation to PASS-Total score (r = 0.202, *p* = 0.01), PASS-FP (r = 0.177, *p* = 0.033), PASS-CA (r = 0.211, *p* = 0.011), and PCS-HP (r = 0.170, *p* = 0.041). The frequency of abortive medication usage was associated with PASS-SA (r = 0.216, *p* = 0.009), PCS-HP (r = 0.176, *p* = 0.035), and PSQ-min (r = 0.181, *p* = 0.029). The duration of migraine illness was associated with PCS-HP (r = 0.180, *p* = 0.030).

Migraine-related disability measured by HIT-6 and MIDAS. The HIT-6 showed a weak correlation with the PASS-Total score (r = 0.390, *p* < 0.001), PASS-FP (r = 0.267, *p* = 0.001), PASS-CA (r = 0.392, *p* < 0.001), PASS-SA (r = 0.301, *p* < 0.001), PASS-EA (r = 0.243, *p* = 0.003), PCS-Total (r = 0.354, *p* < 0.001), PCS-RM (r = 0.354, *p* < 0.001), PCS-MG (r = 0.316, *p* < 0.001), and PCS-HP (r = 0.322, *p* < 0.001). The MIDAS showed a weak correlation with PASS-Total (r = 0.191, *p* = 0.021), PASS-FP (r = 0.181, *p* = 0.030), PASS-CA (r = 0.191, *p* = 0.021), PASS-SA (r = 0.185, *p* = 0.026), and PCS-HP (r = 0.178, *p* = 0.032). The headache characteristics of migraine patients and Pearson correlation results are summarized in Tables 2 and 3.

**Table 2:** headache characteristics of migraine patients.

	Migraine patients (N = 145)
Migraine with aura	5.5%
Duration of illness, years	8.5 ± 7.7
Headache pain intensity (VAS 0–10)	7.6 ± 1.5
Headache frequency (per month)	7.2 ± 6.0
Duration of attack (hours)	35.6 ± 26.8
Presence of nausea or vomiting	89.7%
Presence of photophobia	49.7%
Presence of phonophobia	62.8%
Abortive medication usage (per month)	5.8 ± 4.9
Presence of prophylactic medication	41.4%
HIT-6 score	61.6 ± 6.9
MIDAS score	23.0 ± 36.2

**Table 3:** The relationship between emotional and the cognitive aspect of pain perception and migraine characteristics and headache-related disability.

	PASS					PCS				PSQ		
	Total	FP	CA	SA	EA	Total	RM	MG	HP	Total	min	mod
Duration of illness	0.061	0.024	0.087	− 0.001	0.082	0.137	0.123	0.038	0.180*	0.133	0.134	0.123
Headache pain intensity (VAS)	0.202*	0.177*	0.211*	0.142	0.130	0.132	0.089	0.105	0.170*	0.130	0.142	0.109
Headache frequency	0.062	0.068	0.057	0.157	− 0.094	0.070	0.038	0.025	0.110	0.097	0.131	0.054
Duration of attack	0.054	0.062	0.025	0.088	0.043	0.115	0.060	0.114	0.135	0.023	− 0.003	0.049
Abortive medication	0.121	0.151	0.099	0.216**	− 0.090	0.128	0.074	0.073	0.176*	0.142	0.181*	0.092
HIT-6	0.390**	0.267**	0.392**	0.301**	0.243**	0.354**	0.354**	0.316**	0.322**	0.098	0.085	0.104
MIDAS	0.191*	0.181*	0.191*	0.185*	0.031	0.154	0.082	0.154	0.178*	0.103	0.122	0.077

Pearson’s correlation coefficients and *p* value are shown (*p < 0.05, **p < 0.01).

*FP* fear of pain, *CA* cognitive anxiety, *SA* somatic anxiety, *EA* escape and avoidance, *Rm* rumination, *Mg* magnification, *Hp* helplessness, *Min* minor pain, *Mod* moderate pain.

The multiple regression analyses demonstrated that PASS-Total (*p* = 0.001), HADS-A (*p* = 0.028), and headache frequency (*p* = 0.003) were independently associated with HIT-6. In MIDAS, headache frequency (*p* < 0.001) was identified as independent variables (Table 4).

**Table 4:** Multiple regression analyses for headache-related disability in migraine patients.

	HIT-6				MIDAS			
	B	SE	Partial R^2^	*p* value	B	SE	Partial R^2^	*p* value
PASS-Total	0.1089	0.0323	0.0761	0.0010	0.2926	0.1534	0.0252	0.0585
Headache frequency	0.2699	0.0881	0.0637	0.0026	2.9367	0.4301	0.2485	0.0000
Headache pain intensity (VAS)	0.4992	0.3484	0.0147	0.1541	2.5796	1.7762	0.0147	0.1486
Duration of illness	0.0968	0.0652	0.0157	0.1402				
Duration of attack	0.0352	0.0188	0.0248	0.0631				
HADS-A	0.3102	0.1392	0.0347	0.0275				

**Figure 4:**
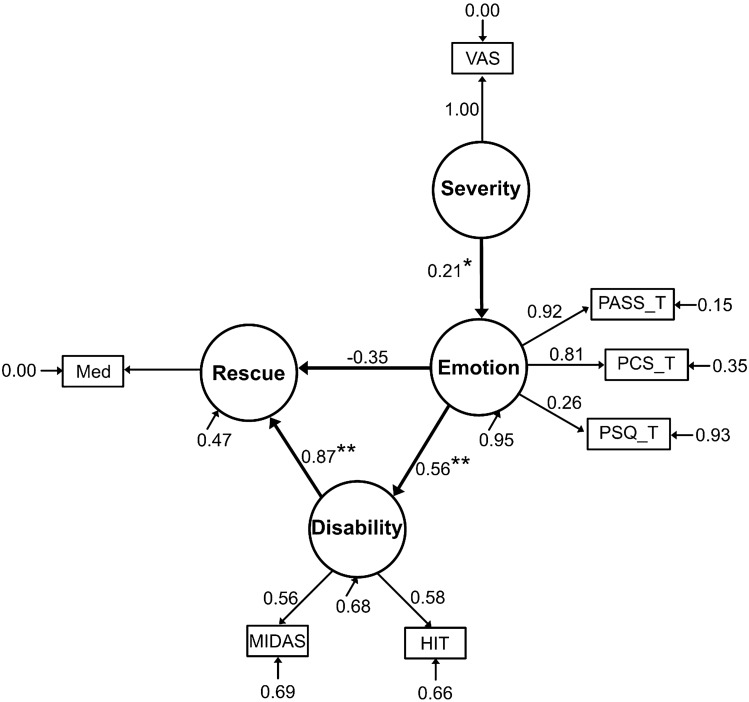
Path diagram of structural equation model with standardized path coefficients. *Note* *p < 0.05; **p < 0.005.

Results of the structural equation model are showed with standardized path estimates (Fig. 4). Model fit indicators show relatively good fit (χ^2^ = 14.14, df = 12, *p* = 0.29, CFI = 0.99, TLI = 0.98, NFI = 0.94, RMSEA = 0.035). Headache severity has significant direct loading (β = 2.30, *p* = 0.014) on pain-related emotion. Pain-related emotion significantly predicts severe headache disability (β = 0.14, *p* < 0.001). Headache disability was associated with abortive medication use (β = 1.05, *p* = 0.002). Pain-related emotion does not have a significant effect on abortive medication use (β = − 0.11, *p* = 0.063).

## Discussion

The major outcomes of the present study were as follows. PASS, PCS, and PSQ scores of migraine patients were significantly higher than those of the control group. PASS and PCS were significantly associated with headache pain intensity, abortive medication use, and headache disability. PASS is one of the important factors contributing to the development of headache-related disability measured by HIT-6 and MIDAS. The structural equation model shows that headache severity leads to a change in the pain-related emotional state. The Pain-related emotion has significant direct loading on headache disability, which is consequently related to rescue usage.

Anxiety or fear for predicting pain can cause different coping strategies, resulting in various perceived pain behaviors. A negative expectation can reverse the analgesic effect, whereas the expectation of pain relief is an important factor for the analgesic effect of a placebo^27,28^. The emotional and psychological problems could exacerbate a headache in migraine^29,30^. The neuronal network for pain and emotion are overlapped and have a mutual influence on each other^31,32^. The periaqueductal gray (PAG) and rostral ventromedial medullar play an important role in migraine pathogenesis and are closely related to various parts of the brain, including the amygdala, hippocampus, orbitofrontal cortex, and insula, which are responsible for pain-related cognition and emotion^29,33,34^. These structural connections indicate that psychological factors may affect pain modulation in migraine patients.

The emotional contributions for pain were reported in various chronic pain disorders, including back pain, cancer pain, rheumatoid arthritis, and tissue injury by using PASS, PCS, and PSQ^14,26,35^. The PCS score related to fear of pain, frequency, chronicity, and headache severity in pediatric headache and obese women with migraine^15,36^. In the headache patients, anxiety sensitivity associated with fear of pain and escape or avoidance^3^.

### The alteration of pain-related emotion in migraine

The PASS score used to evaluate pain-related anxiety, and the score over 30 suggests the problematic pain-related anxiety in young adults without current and chronic pain^21^. Other studies set the cut-off value of total PASS score: mild (0–33), moderate (34–67), and severe pain anxiety (68–100)^37^. The present study showed that migraine sufferers have a range of moderate to severe pain-related anxiety, according to the PASS score (47.4 ± 17.2). It is known that anxiety is significantly associated with an increased risk of migraine^30,38^. The PCS is a tool for evaluating catastrophizing chronic pain disorders. A total PCS score over 30 is associated with clinically significant catastrophizing^14^. Previous studies reported that the catastrophized group showed more emotional distress and severe pain intensities than the groups of the normal and chronic pain group without catastrophizing^14,39,40^. In our study, the migraine patients had a higher PCS score (24.4 ± 14.1) than the control group, even though it did not reach a clinically significant catastrophizing level. PSQ evaluates the pain perception based on the imagined painful daily situation. The PSQ-min and total scores were significantly higher than the control in chronic pain diseases such as chronic tension-type headache, chronic low back pain, chronic mandibular pain, and chronic mixed pain disorder^13^. Moreover, PSQ related to headache pain intensity, catastrophizing, and central sensitization^13,41^. In the present study, the migraine patient group had a higher PSQ score than the control, similar to previous studies^13,41^.

We consider the possibility that migraine patients exhibited an altered emotional and response to a pain similar to other chronic pain disorders.

### The relationship between migraine characteristics, disability, and pain-related emotion

The correlation analysis showed that pain-related anxiety, especially FP and CA, weakly correlated to headache pain intensity in migraine patients. PASS-FP was intended to measure fearful thoughts related to the experience of pain or the anticipated negative consequences of pain. PASS-CA was to assess cognitive symptoms associated with the experience of pain, such as racing thoughts or impaired concentration. Although the results vary depending on the study, subdomains of PASS related to the severity of pain are reported^42–44^. Pain severity was associated with PASS-FP, CA, and SA in chronic back pain^43^. Other studies reported that all PASS subdomains were associated with pain severity in heterogeneous chronic pain patients^42^. In headache patients, headache severity had direct loading on all PASS subdomains^3^. Interestingly, the present study suggested that PASS-SA was related to the behavioral consequence of taking abortive medication for headache attacks. PASS-SA assessed symptoms reflecting physiological arousal associated with the experience of pain. At the start of the study, we assumed that PASS-EA was probably associated with abortive medication use because PASS-EA contains an item to take medication to avoid pain. However, many other items of PASS-EA also include directions to reduce or stabilize activity to avoid pain, which may dilute the possibility of medication use. From the present study, we can assume that abortive medication could have priority when a physical discomfort is noticeable in migraine patients.

The PCS-HP weakly correlates with headache duration, headache pain intensity, and abortive medication use, which were similar to in a previous study suggesting the relationship between headache pain intensity, duration, and frequency in obese women with migraine^36^. As migraine becomes severe and more frequent, it could be considered that the feeling of helplessness may increase when in pain. The present study showed significant but weak correlations between pain-related emotion and migraine characteristics. Therefore, a careful approach is needed to interpret our correlation analysis results.

The present study showed that PASS, headache frequency, and HADS-A has a significant contribution to HIT-6 in multiple regression analysis. Considering that the main factor determining the MIDAS score is the frequency of the headache itself, the relative contribution of PASS can be meaningful enough. The structural equation model showed that headache severity enhances the headache-related emotion and has significant loading to migraine-related disability. The PASS score significantly correlated to the pain-related disability in chronic back pain and musculoskeletal disorder^37,43^. McCracken et al. reported that the PASS score showed a significant correlation and is predictive of disability in various chronic pain disorders^7^. Another study reported that pain-related anxiety is a unique predictor of headache disability using PASS and MIDAS in migraine and tension-type headache^22^. In patients with chronic migraine, depression, anxiety, and catastrophizing were associated with severe migraine-related disability^45^. These studies suggest that the headache-related emotion, especially anxiety, is highly related to migraine-related disability. From the structural equation model, we could better understand the causal link between headache-related anxiety and disability in migraine patients.

Our result showed that the use of abortive medication was directly related to disability more than the emotional factor. The fear of pain influences avoidance behavior in headache patients^3^. The correlation between avoidance and pain-related disability is reported in various chronic pain disorders^46,47^. It seems appropriate to understand that abortive medication usage in migraine is an action that reflects active treatment-seeking behavior rather than an avoidance of pain.

In conclusion, the present study showed that migraine patients had altered emotional and cognitive responses to pain perception. The contributions of PASS to HIT-6 and MIDAS highlight the effect of distorted pain perception on headache, especially anxiety, and may be related to disability in migraine. In the therapeutic aspect of migraines, the strategy for disability considering pain-related emotional factors could be important. Further study is also needed to analyze a clear temporal relationship between pain-related emotional factor and disability in migraine.

This study is limited in several ways. The present research was based on a one-time point evaluation. Thus, we were unable to explain the causal relationship and examine test–retest reliability clearly. By analyzing a single model, the proposed structural equation model may not be perfect. Many prophylactic medications can directly influence the emotional status. In this study design, we did not properly clarify this issue. Further study would be necessary to conduct with the factors of prophylactic medication as an important variable. We lacked information and knowledge on behavioral factors that can affect individual pain perception, that is, the degree of social activity engagement, interpersonal relationship, and work-related activities.

## References

[1] Stovner LJ, Hoff JM, Svalheim S, Gilhus NE (2014). Neurological disorders in the Global Burden of Disease 2010 study. Acta Neurol. Scand. Suppl..

[2] D'Antona L, Matharu M (2019). Identifying and managing refractory migraine: barriers and opportunities?. J. Headache Pain.

[3] Norton PJ, Asmundson GJ (2004). Anxiety sensitivity, fear, and avoidance behavior in headache pain. Pain.

[4] Edwards RR (2005). Individual differences in endogenous pain modulation as a risk factor for chronic pain. Neurology.

[5] Roth RS, Geisser ME, Theisen-Goodvich M, Dixon PJ (2005). Cognitive complaints are associated with depression, fatigue, female sex, and pain catastrophizing in patients with chronic pain. Arch. Phys. Med. Rehabil..

[6] Lethem J, Slade PD, Troup JD, Bentley G (1983). Outline of a fear-avoidance model of exaggerated pain perception—I. Behav. Res. Ther..

[7] McCracken LM, Zayfert C, Gross RT (1992). The Pain Anxiety Symptoms Scale: development and validation of a scale to measure fear of pain. Pain.

[8] Crombez G, Vlaeyen JW, Heuts PH, Lysens R (1999). Pain-related fear is more disabling than pain itself: evidence on the role of pain-related fear in chronic back pain disability. Pain.

[9] Williams DA, Keefe FJ (1991). Pain beliefs and the use of cognitive-behavioral coping strategies. Pain.

[10] French DJ (2000). Perceived self-efficacy and headache-related disability. Headache.

[11] Cho S, Lee S-M, McCracken LM, Moon D-E, Heiby EM (2010). Psychometric properties of a Korean version of the Pain Anxiety Symptoms Scale-20 in chronic pain patients. Int. J. Behav. Med..

[12] McCracken LM, Faber SD, Janeck AS (1998). Pain-related anxiety predicts non-specific physical complaints in persons with chronic pain. Behav. Res. Ther..

[13] Ruscheweyh R (2012). Validation of the pain sensitivity questionnaire in chronic pain patients. Pain.

[14] Sullivan MJL, Bishop SR, Pivik J (1995). The Pain Catastrophizing Scale: development and validation. Psychol. Assess..

[15] Simons LE, Pielech M, Cappucci S, Lebel A (2015). Fear of pain in pediatric headache. Cephalalgia.

[16] Park JW, Cho SJ, Park SG, Chu MK (2018). Circadian variations in the clinical presentation of headaches among migraineurs: a study using a smartphone headache diary. Chronobiol. Int..

[17] Park JW, Chu MK, Kim JM, Park SG, Cho SJ (2016). Analysis of trigger factors in episodic migraineurs using a smartphone headache diary applications. PLoS ONE.

[18] Headache Classification Committee of the International Headache Society (IHS) (2013). The International Classification of Headache Disorders, 3rd edition (beta versiom). Cephalalgia.

[19] Faul F, Erdfelder E, Buchner A, Lang AG (2009). Statistical power analyses using G*Power 3.1: tests for correlation and regression analyses. Behav. Res. Methods.

[20] Faul F, Erdfelder E, Lang AG, Buchner A (2007). G*Power 3: a flexible statistical power analysis program for the social, behavioral, and biomedical sciences. Behav. Res. Methods.

[21] Abrams MP, Carleton RN, Asmundson GJ (2007). An exploration of the psychometric properties of the PASS-20 with a nonclinical sample. J. Pain.

[22] Nash JM, Williams DM, Nicholson R, Trask PC (2006). The contribution of pain-related anxiety to disability from headache. J. Behav. Med..

[23] Cho S, Kim HY, Lee JH (2013). Validation of the Korean version of the Pain Catastrophizing Scale in patients with chronic non-cancer pain. Qual. Life Res..

[24] Kim HJ (2014). Translation, cross-cultural adaptation, and validity of the Korean version of the pain sensitivity questionnaire in chronic pain patients. Pain Pract..

[25] Osman A (1997). Factor structure, reliability, and validity of the Pain Catastrophizing Scale. J. Behav. Med..

[26] Ruscheweyh R, Marziniak M, Stumpenhorst F, Reinholz J, Knecht S (2009). Pain sensitivity can be assessed by self-rating: Development and validation of the Pain Sensitivity Questionnaire. Pain.

[27] Bingel U (2011). The effect of treatment expectation on drug efficacy: imaging the analgesic benefit of the opioid remifentanil. Sci Transl Med.

[28] Benedetti F, Mayberg HS, Wager TD, Stohler CS, Zubieta JK (2005). Neurobiological mechanisms of the placebo effect. J. Neurosci..

[29] Nicholson RA, Houle TT, Rhudy JL, Norton PJ (2007). Psychological risk factors in headache. Headache.

[30] Peres MFP, Mercante JPP, Tobo PR, Kamei H, Bigal ME (2017). Anxiety and depression symptoms and migraine: a symptom-based approach research. J. Headache Pain.

[31] Prakash S, Golwala P (2011). Phantom headache: pain-memory-emotion hypothesis for chronic daily headache?. J. Headache Pain.

[32] Bushnell MC, Ceko M, Low LA (2013). Cognitive and emotional control of pain and its disruption in chronic pain. Nat. Rev. Neurosci..

[33] Lazarov NE (2011). Amygdalotrigeminal projection in the rat: an anterograde tracing study. Ann. Anat..

[34] Akerman S, Holland PR, Goadsby PJ (2011). Diencephalic and brainstem mechanisms in migraine. Nat. Rev. Neurosci..

[35] McCracken LM, Dhingra L (2002). A short version of the Pain Anxiety Symptoms Scale (PASS-20): preliminary development and validity. Pain Res. Manag..

[36] Bond DS (2015). Clinical pain catastrophizing in women with migraine and obesity. Headache.

[37] Brede E, Mayer TG, Neblett R, Williams M, Gatchel RJ (2011). The Pain Anxiety Symptoms Scale fails to discriminate pain or anxiety in a chronic disabling occupational musculoskeletal disorder population. Pain Pract..

[38] Farris SG (2019). Anxiety sensitivity as a risk indicator for anxiety, depression, and headache severity in women with migraine. Headache.

[39] Sullivan MJ, Stanish W, Waite H, Sullivan M, Tripp DA (1998). Catastrophizing, pain, and disability in patients with soft-tissue injuries. Pain.

[40] Holroyd KA, Drew JB, Cottrell CK, Romanek KM, Heh V (2007). Impaired functioning and quality of life in severe migraine: the role of catastrophizing and associated symptoms. Cephalalgia.

[41] Tuna T, Van Obbergh L, Van Cutsem N, Engelman E (2018). Usefulness of the pain sensitivity questionnaire to discriminate the pain behaviour of chronic pain patients. Br. J. Anaesth..

[42] Coons MJ, Hadjistavropoulos HD, Asmundson GJ (2004). Factor structure and psychometric properties of the Pain Anxiety Symptoms Scale-20 in a community physiotherapy clinic sample. Eur. J. Pain.

[43] Vowles KE, Zvolensky MJ, Gross RT, Sperry JA (2004). Pain-related anxiety in the prediction of chronic low-back pain distress. J. Behav. Med..

[44] Strahl C, Kleinknecht RA, Dinnel DL (2000). The role of pain anxiety, coping, and pain self-efficacy in rheumatoid arthritis patient functioning. Behav. Res. Ther..

[45] Seng EK (2017). Psychological factors associated with chronic migraine and severe migraine-related disability: an observational study in a tertiary headache center. Headache.

[46] Andrews NE, Strong J, Meredith PJ (2012). Activity pacing, avoidance, endurance, and associations with patient functioning in chronic pain: a systematic review and meta-analysis. Arch. Phys. Med. Rehabil..

[47] Hasenbring MI, Hallner D, Rusu AC (2009). Fear-avoidance- and endurance-related responses to pain: development and validation of the Avoidance-Endurance Questionnaire (AEQ). Eur. J. Pain.

